# Decreased Appetite is Associated with Sarcopenia-Related Outcomes in Acute Hospitalized Older Adults

**DOI:** 10.3390/nu11040932

**Published:** 2019-04-25

**Authors:** Carliene van Dronkelaar, Michael Tieland, Jesse J. Aarden, Lucienne A. Reichardt, Rosanne van Seben, Marike van der Schaaf, Martin van der Esch, Raoul H. H. Engelbert, Jos W. R. Twisk, Jos A. Bosch, Bianca M. Buurman

**Affiliations:** 1 Faculty of Sports and Nutrition, Amsterdam University of Applied Sciences, 1067SM Amsterdam, The Netherlands; m.tieland@hva.nl; 2 Department of Rehabilitation, Amsterdam Movement Sciences, Amsterdam UMC, University of Amsterdam, 1105 AZ Amsterdam, The Netherlands; j.j.aarden@hva.nl (J.J.A.); m.vanderschaaf@amc.uva.nl (M.v.d.S.); 3 Faculty of Health, Amsterdam University of Applied Sciences, ACHIEVE-Center of Applied Research, 1105 BD Amsterdam, The Netherlands; m.vd.esch@reade.nl (M.v.d.E.); r.h.h.engelbert@hva.nl (R.H.H.E.); b.m.vanes@amc.uva.nl (B.M.B.); 4 Department of Internal Medicine, Section of Geriatric Medicine, Amsterdam Public Health Research Institute, Amsterdam UMC, University of Amsterdam, 1105 AZ Amsterdam, The Netherlands; l.a.reichardt@amc.uva.nl (L.A.R.); r.vanseben@amc.uva.nl (R.v.S.); 5 Reade, Center for Rehabilitation and Rheumatology/Amsterdam Rehabilitation Research Center, 1056 AB Amsterdam, The Netherlands; 6 Department of Epidemiology and Biostatistics, Amsterdam UMC, Vrije Universiteit Amsterdam, 1081 HV Amsterdam, The Netherlands; jwr.twisk@vumc.nl; 7 Department of Clinical Psychology, University of Amsterdam, 1018 WS Amsterdam, The Netherlands; j.a.bosch@uva.nl; 8 Department of Medical Psychology, Amsterdam UMC, University of Amsterdam, 1105 AZ Amsterdam, The Netherlands

**Keywords:** nutrition, malnutrition, post-acute care, muscle strength, muscle mass, mobility, physical performance

## Abstract

Decreased appetite is one of the main risk factors of malnutrition. Little is known on how appetite changes during hospitalization and after discharge and how it relates with sarcopenia-related outcomes. We analyzed data of the Hospital-ADL study, a multicenter prospective cohort study that followed 400 acutely hospitalized older adults (≥70 year). Appetite (SNAQ), handgrip strength (Jamar), muscle mass (BIA), mobility (DEMMI), and physical performance (SPPB) were assessed within 48 h of admission, at discharge, and at one and three months post-discharge. The course of decreased appetite was analysed by Generalised Estimating Equations. Linear Mixed Model was used to analyse the associations between decreased appetite and the sarcopenia-related outcomes. Decreased appetite was reported by 51% at hospital admission, 34% at discharge, 28% one month post-discharge, and 17% three months post-discharge. Overall, decreased appetite was associated with lower muscle strength (β = −1.089, *p* = 0.001), lower mobility skills (β = −3.893, *p* < 0.001), and lower physical performance (β = −0.706, *p* < 0.001) but not with muscle mass (β = −0.023, *p* = 0.920). In conclusion, decreased appetite was highly prevalent among acute hospitalized older adults and remained prevalent, although less, after discharge. Decreased appetite was significantly associated with negative sarcopenia-related outcomes, which underlines the need for assessment and monitoring of decreased appetite during and post hospitalization.

## 1. Introduction

As society ages, the number of people with limitations in physical function will increase [1,2,3,4]. A major cause of these physical limitations is the age-related low muscle mass, strength, and physical function, also known as sarcopenia [5,6]. These sarcopenia-related outcomes decline 1–4% per year, which may be accelerated during hospitalization [7,8]. Notably, sarcopenic hospitalized older adults have a high risk for adverse health outcomes, such as increased hospital stay, readmissions, and mortality [9,10,11]. Therefore, screening, treatment, and prevention of sarcopenia is of great importance for improving patients’ quality of life and reducing health care costs [9,12]. 

A major risk factor for hospital related sarcopenia is malnutrition of which prevalence rates among hospitalized older adults can be as high as 38% [5,8,13,14,15,16]. In these patients, a decreased appetite is the primary cause of malnutrition. [17,18,19]. The prevalence of a decreased appetite is reported by 64% during hospitalization and by 28% after discharge [15,20,21,22]. Yet, very limited data is available on the course of changes in decreased appetite during and post hospitalization. Pilgrim et al. showed that 52% of the older women that reported a decreased appetite at hospital admission still had a decreased appetite six months after discharge [15]. However, although the first three months after discharge are critical for recovery, little is known on how appetite changes during hospitalization and three months after discharge [23,24]. In addition, very little information is available on how a decreased appetite relates to sarcopenia-related outcomes such as muscle strength, muscle mass, mobility, and physical performance during and after hospitalization [5,22]. Therefore, the aim of this study is to assess (1) the course of decreased appetite during acute hospitalization as well as one to three months post hospital discharge and (2) the association between decreased appetite and muscle mass, muscle strength, mobility, and physical performance during and post hospitalization in older adults.

## 2. Materials and Methods

### 2.1. Subjects and Design

Data from the Hospital-Associated Disability and impact on daily Life study (Hospital-ADL study), a multicenter, observational, prospective cohort study, was used. The study protocol is described elsewhere [25]. In short, 401 acutely hospitalized older adults admitted for at least 48 h, aged ≥70, Mini-Mental State Examination score ≥15, were recruited from departments of Internal Medicine, Cardiology or Geriatrics from six Dutch teaching and community hospitals. The aim was to investigate cognitive, behavioral, psychosocial, physical, and biological factors that may be associated with hospitalization-associated disability from hospital admission to three months post-discharge. The Hospital-ADL study was approved by the Institutional Review board of the Academic Medical Center (AMC) in the Netherlands (Protocol ID: AMC2015_150). Written informed consent was obtained from all subjects before inclusion.

### 2.2. Data Collection 

All variables were measured at the following time points: within 48 h of hospital admission, at hospital discharge, at one month and three months post-discharge.

#### 2.2.1. Decreased Appetite

The Short Nutritional Assessment Questionnaire was used to identify malnourished hospital patients [17]. One of the questions of this questionnaire was on self-reported appetite, namely “Have you experienced a decreased appetite over the last month?” and was answered with “yes” or “no”. This question was adjusted for discharge measurement to “Have you experienced a decreased appetite since hospital admission?”.

#### 2.2.2. Muscle Strength

Muscle strength was measured by handgrip strength (HGS; Jamar grip strength dynamometer; Lafayette Instrument Company, USA) [26]. Subjects were measured in supine or sitting position and encouraged to show maximal isometric handgrip strength. The scores were noted in kilograms after HGS was performed three times for each hand. The maximum score for either hand was used for analyses.

#### 2.2.3. Muscle Mass

Skeletal Muscle Mass (SMM) was assessed by bio-impedance analysis (BIA; Bodystat Quascan 4000) [27]. Subjects were asked to assume a supine position with arms not touching the trunk and legs or feet not touching each other. Injection electrodes were placed wrist-to-ankle and sensing electrodes placed hand-to-foot on the ipsilateral side of the body. A small electrical signal circulated which measured the resistance of this electrical signal from which the SMM was estimated based on the equation of Janssen et al. [28]. Patients’ SMM in kilograms was used for analysis.

#### 2.2.4. Mobility

In this paper, mobility was defined as a measure of how well one can move [4,29]. The Dutch version of de Morton Mobility Index (DEMMI), a 15 item unidimensional measure based on Rasch analysis, was used to assess mobility [29]. Subjects were asked to perform the following tasks: perform a bridge, roll onto side, lie to sit, sit unsupported in chair, sit to stand from chair, sit to stand without using arms, stand unsupported, stand feet together, stand on toes, tandem stand, walking distance, walking assistance, pick up pen from floor, walk 4 steps backwards, and jump. The tasks were scored according the standardized protocol. The raw score was converted into an interval-level score out of 100. A higher score means a better mobility performance and a change of 10 points is reported as the minimally clinically important difference [30].

#### 2.2.5. Physical Performance

In this paper, physical performance was defined as a measure of endurance of specific movements [4]. Physical performance was measured by the Short Physical Performance Battery (SPPB) [31]. The SPPB measures balance, strength, and gait speed. Subjects were asked to stand with their feet in a side-by-side position, a semi-tandem position, a full-tandem position, walk a distance of four meters, and to rise from a chair and return to the seated position five times as quickly as possible. The total score ranges from 0 to 12 points. A meaningful change in SPPB score ranges from 0.5 to 1.0 point [32,33].

#### 2.2.6. Other Variables

Data on age, gender, education, ethnicity, living situation, marital status, cognitive impairment (Mini Mental State Examination (MMSE), score ≤23) [34], depression (Geriatric Depression Scale-15 (GDS-15) score ≥6) [35], activities of daily living (Modified Katz Index Scale-6 (ADL-KATZ6)) [36], fatigue (NRS fatigue) [37], fear of falling (NRS fear of falling), reason for initial admission, comorbidity (Charlson Comorbidity Index(CCI)) [38], length of hospital stay (LOS), malnutrition (Short Nutritional Assessment Questionnaire (SNAQ), score ≥3) [17] and readmission were collected.

### 2.3. Statistical Analysis

Data was analyzed using Statistical Package for Social Science (SPSS) version 24 (SPSS Inc., Chicago, IL, USA). Descriptive statistics were used to summarize the patient demographic and medical characteristics. To assess the course of appetite, Generalized Estimating Equations (GEE) analyses were performed. The obtained odds ratios (ORs) represent the odds for finding a similar prevalence rate for a decreased appetite at discharge, one or three months post-discharge as compared to the prevalence rate at admission and indicates whether prevalence rates significantly increase or decrease. To test the association between decreased appetite (independent variable) and muscle strength, muscle mass, mobility, and physical performance (dependent variables) over time, Linear Mixed Model (LMM) analyses were performed [39]. Data was checked on normality by plotting histograms of the residuals. All data was normally distributed. Both GEE and LMM take into account the correlation between the repeated observations. GEE is more suitable to analyze dichotomous outcomes, while LMM is more suitable to analyze continuous outcomes [39,40]. To identify effect modification of gender, age, fatigue, and depression, interaction terms between decreased appetite and these variables were added to the crude model. For the LMM analyses, first an overall analysis was performed, taking the average of all timepoints. Second, the hospitalization period was compared with the post discharge period. Hospitalization was the average of the time points at admission and at discharge. For the post-discharge period the average of the timepoints one month post-discharge and three months post-discharge was used. Third, every timepoint was analyzed separately. Besides a crude analysis, also adjusted analyses were performed. The potential confounders were based on literature [41,42,43,44]. All parameter estimates were expressed with a 95% confidence interval (95% CI). Statistical significance was set at *p* < 0.05.

## 3. Results

### 3.1. Study Sample Characteristics

Between October 2015 and February 2017, 1024 acutely hospitalized patients were approached, of which 519 met the inclusion criteria and 401 agreed to participate. One patient did not have data available on decreased appetite at baseline. Therefore, data of 400 patients were included in the analytical sample. During the study, 84 patients were lost to follow up, four patients became terminally ill, and 40 patients died. Patients’ baseline characteristics are provided in Table 1. Overall, 51.5% of the participants were men and the mean (SD) age was 80.1 (6.68) years.

### 3.2. The Course of Decreased Appetite

At admission, 51% of the subjects reported a decreased appetite. The subjects who reported to have a decreased appetite were more often women, had a longer length of stay, more comorbidities, more often polypharmacy, scored higher on fatigue and the geriatric depression scale, had more trouble to perform activities of daily living, were more often malnourished, had lower handgrip strength, lower skeletal muscle mass, and scored lower on mobility and physical performance (Table 1).

Of those who had a decreased appetite at admission, 21% still had decreased appetite three months after discharge. The prevalence rates of decreased appetite were significantly lower at discharge, one and three months post-discharge compared to admission. The odds ratios hardly changed when analyses were repeated using only complete cases on decreased appetite (Table 2).

### 3.3. Decreased Appetite and Muscle Strength

An inverse association was found between decreased appetite and muscle strength (crude model; β = −0.73, *p* = 0.005). This association became stronger, when the crude model was adjusted for age, gender, cognitive impairment, fatigue, depression, comorbidity, and lean body mass (β = −1.09, *p* = 0.001). This means that subjects who reported to have a decreased appetite performed lower on hand grip strength by 1.09 kg compared to the subjects who reported to not have a decreased appetite. No effect modification with age, gender, fatigue or depression was found. Considering the specific time periods during hospitalization and post-discharge, a significant association was found between decreased appetite and muscle strength in both periods (adjusted model; β = −0.84, *p* = 0.030 and β = −1.71, *p* < 0.001 respectively). Analyses at every time point showed that these associations were most pronounced at three months post-discharge (adjusted model; β = −2.97, *p* < 0.001) (Table 3).

### 3.4. Decreased Appetite and Skeletal Muscle Mass

There was no significant association between decreased appetite and skeletal muscle mass in both the crude and adjusted model (β = −0.09; *p* = 0.712 and β = −0.02; *p* = 0.920 respectively). Also no effect modification with age, gender, fatigue, or depression was found. Analyses at the different time points showed similar results (Table 3).

### 3.5. Decreased Appetite and Mobility

There was a significant inverse association between decreased appetite and mobility (crude model; β = −6.45; *p* < 0.001). After adjustment, this association became less strong (β = −3.89; *p* < 0.001). This association means that subjects who reported to have a decreased appetite scored 3.89 points lower on the DEMMI compared to the subjects who reported to not have a decreased appetite. No effect modification with age, gender, fatigue or depression was found. Decreased appetite was significantly associated with lower mobility skills during hospitalization (adjusted model; β = −3.98, *p* = 0.003). In addition, further analyses at every time point showed that this association was most pronounced (adjusted model; β = −4.15, *p* = 0.012) (Table 3).

### 3.6. Decreased Appetite and Physical Performance

There was a significant inverse association between decreased appetite and physical performance (crude model; β = −1.08, *p* < 0.001). This association remained significant after adjustment, but became less strong (β = −0.71, *p* < 0.001). This association means that subjects who reported to have a decreased appetite scored 0.71 points lower on the SPPB compared to the subjects who reported to not have a decreased appetite. No effect modification of age, gender, fatigue, or depression was found. The association was found to be present during hospitalization (adjusted model; β = −0.56, *p* = 0.017). Analyses at every time point showed that the significant association was most pronounced at one month post-discharge (adjusted model; β = −0.79, *p* = 0.019) (Table 3).

## 4. Discussion

This multicenter prospective cohort study showed that decreased appetite is highly prevalent among acute hospitalized older adults and remained prevalent, although less, three months post-discharge. Overall, decreased appetite was associated with lower muscle strength, mobility, and physical performance but not with muscle mass. In more detail, at admission, an association between decreased appetite and lower muscle strength and mobility was found. At one month post-discharge there was an association between decreased appetite and lower physical performance. At three months after discharge, an association between decreased appetite and lower muscle strength was found. 

At admission, a prevalence rate of a decreased appetite was reported by 51% of the subjects. Although our prevalence rates are in line with some studies, Pilgrim et al. did show a somewhat higher prevalence rate (38%) six months post-discharge [15,22,45]. This difference might be due to the fact that our study had subjects who had a shorter length of stay and decreased appetite was reported in a shorter time after discharge. In another population, Tsaousi et al. showed that impaired appetite was an important determinant for predicting length of hospital stay [46]. A longer length of hospital stay in malnourished patients is reported by several studies [14,47,48]. Decreased appetite is one of the main risk factors for malnutrition [17,18,19]. In line with these studies, our study showed that subjects with a decreased appetite at admission had a significantly longer length of hospital stay. 

Subjects who reported decreased appetite at admission scored higher on depression and fatigue. Depression is often associated with changes in appetite, whereas this has not been shown for fatigue yet [42,49]. In our study, we checked for effect modification of depression and fatigue, however this was not the case. 

To our knowledge, this study is the first to assess the longitudinal association between decreased appetite and sarcopenia-related outcomes over the time span from hospital admission to three months post-discharge. The study of Pilgrim et al. reported an association of decreased appetite with lower handgrip strength at hospital admission in older female patients, which is in line with our findings [15]. Furthermore, Reijnierse et al. reported an association of decreased appetite with diagnostic measures of sarcopenia in geriatric outpatients, showing similar results for handgrip strength, but not for muscle mass [22]. This discrepancy can be explained by the differences in the study populations. A meta-analyses of Van Ancum et al. reported that acutely hospitalized patients do not show a decline in skeletal muscle mass, whereas in elective hospitalized patients there was a decline in muscle mass [50]. It is possible that in acutely hospitalized patients there was already a decline in muscle mass before hospitalization and therefore no decline showed post-discharge. Furthermore, length of stay was relatively short in our study in comparison with bed rest studies, which may explain the lack of change in skeletal muscle mass that was observed during hospitalization [7]. Also, skeletal muscle mass was assessed with a non-segmental BIA. A BIA measurement can be influenced by hydration status [51]. The use of BIA to measure skeletal muscle mass can give an underestimation of the change in muscle mass as patients may be dehydrated at admission and the increase in fluid may mask the change in muscle mass [51,52]. No information on hydration status was available in this study. However, BIA is still the most feasible and non-invasive method in use for this older and frail patient group as dual-energy X-ray absorptiometry (DXA), which would be the preferred instrument, is not yet a portable device [5,53]. 

In our study, decreased appetite is associated with lower mobility skills and lower physical performance during hospitalization. Terminology of mobility and physical performance are used interchangeable or even taken all together with muscle strength in physical function [53]. In addition, different tests are used to assess mobility and/or physical performance which makes it difficult to compare studies on a detailed level. In our study, we defined mobility as a measure of how well one can move, whereas physical performance was defined as a measure of endurance of specific movements [4,29]. At admission, Pilgrim et al. reported an association between poor appetite and a lower Barthel Index score, which is a questionnaire on activities of daily living [15,54]. The study of Reijnierse et al. found no association between decreased appetite and lower physical performance, measured with walking speed, in geriatric outpatients [22]. Our longitudinal associations showed comparable results. 

Decreased appetite is part of the SNAQ screening tool to assess patients at risk of malnutrition [17]. Following the newest Global Leadership Initiative on Malnutrition (GLIM) criteria, screening for patients at risk for malnutrition is the key first step in evaluation of nutrition status [55]. This study underlines the need for assessment of decreased appetite in the screening tool as it is associated with lower muscle strength, lower mobility skills, and lower physical performance in acutely hospitalized older patients. Furthermore, decreased appetite should be monitored during the course of hospitalization up to at least three months after discharge as it is still prevalent. In addition, interventions targeting sarcopenia in acutely hospitalized older adults should consider addressing decreased appetite and should be performed both during and after hospitalization.

To our best knowledge, this study is one of the first to show the course of decreased appetite from acute hospitalization to three months after discharge. A strength of this study is its multicenter longitudinal design with repeated measures in both teaching and community hospitals. The measurements were performed in a structural and protocolled manner [25]. In addition, we were able to include 400 acutely hospitalized older adults in the study. However, some limitations need to be addressed. Firstly, due to the observational setting of the study, no causality could be studied. Secondly, 31% of the patients were lost to follow up or passed away within three months post-discharge. It is plausible that patients who were lost to follow-up may have had a decreased appetite. Therefore, we performed a sensitivity analysis on the prevalence of decreased appetite, which yielded similar results. In addition, the analyses on the associations were performed with linear mixed models, which accounts for missing data [39]. Thirdly, no information was available on food intake. A decreased appetite could lead up to a reduced food intake [45]. However, reduced food intake did not seem to be a predictor of the development of malnutrition within the development of the SNAQ tool [17]. Also, when reduced food intake is associated with poor appetite it is unlikely that food intake will increase by simple provision of oral supplements [45]. Therefore, it seems reasonable to assess decreased appetite in the context of malnutrition.

## 5. Conclusions

Decreased appetite was highly prevalent among acute hospitalized older adults and remained prevalent, although less, after discharge. Decreased appetite was associated with handgrip strength and physical function but not with skeletal muscle mass. Further research should focus on how to improve appetite and how to reduce malnutrition in hospitalized older adults and study its effects on muscle strength, mobility, and physical performance.

## Figures and Tables

**Table 1:** Baseline characteristics of study sample.

	All (*n* = 400)	No decreased appetite at admission (*n* = 198)	Decreased appetite at admission (*n* = 202)
Age, median (IQR)	79.5 (74.6–85.1)	78.7 (74.5–85.0)	80.0 (75.1–85.5)
Gender, male, *n* (%)	206 (51.5)	115 (58.1)	91 (45.0) *
BMI, kg/m^2^, median (IQR)	24.5 (21.9–28.6)	24.8 (22.9–28.7)	24.3 (21.2–28.1)
Reason for admission, *n* (%)			
Infection	58 (14.5)	32 (16.2)	26 (12.9)
Gastrointestinal	45 (11.3)	19 (9.6)	26 (12.9)
Cardiac	121 (30.3)	64 (32.3)	57 (28.2)
Respiratory	75 (18.8)	36 (18.2)	39 (19.3)
Cancer (including haematology)	13 (3.3)	5 (2.5)	8 (4.0)
Electrolyte disturbance	11 (2.8)	4 (2.0)	7 (3.5)
Renal	15 (3.8)	9 (4.5)	6 (3.0)
Other	62 (15.5)	29 (14.6)	33 (16.3)
Living arrangement before admission, *n* (%)			
Independent	337 (84.3)	170 (85.8)	167 (82.7)
Nursing home	8 (2.1)	3 (1.5)	5 (2.5)
Senior residence/Assisted living	55 (13.8)	25 (3.5)	30 (14.9)
Length of stay, days, median (IQR)	5.8 (3.9–8.9)	5.2 (3.4–7.9)	6.6 (3.9–11.5) *
Level of education, *n* (%)			
Primary school	101 (25.3)	48 (24.2)	53 (26.2)
Elementary technical/domestic science school	88 (22.0)	43 (21.7)	45 (22.3)
Secondary vocational education	120 (30.0)	72 (36.4)	79 (39.1)
Higher level high school/third-level education	91 (22.8)	35 (17.7)	25 (12.4)
CCI, median (IRQ)	2 (1–3)	1 (1–3)	2 (1–4) *
Polypharmacy, n (%)	259 (64.8)	118 (59.6)	141 (69.8) *
GDS-15, median (IQR)	3 (2–5)	3 (1–4)	4 (2–6) *
MMSE, median (IQR)	27 (24–28)	27 (25–28)	27 (24–28)
ADL-KATZ6, median (IQR)	1 (0–3)	1.0 (0.0–2.0)	1.5 (0.0–3.0) *
Fatigue, median (IQR)	6 (4–8)	6 (3–7)	7 (5–8) *
Malnourished, n (%)	132 (33.0)	17 (8.6)	115 (56.9) *
Handgrip strength, kg, median (IQR)	26 (20–34)	28 (21–36)	24 (18–32) *
Skeletal muscle mass, kg, median (IQR)	22.7 (17.5–28.1)	24.8 (18.7–29.5)	21.8 (17.3–26.9) *
DEMMI, median (IQR)	57 (41–74)	62 (44–74)	53 (39–67) *
SPPB, median (IQR)	5.0 (2.0–8.0)	6.5 (3.0–9.0)	4.0 (2.0–7.0) *

SD = Standard Deviation; IQR = Inter Quartile Range; BMI = Body Mass Index; CCI = Charlson Comorbidity Index (higher score indicating a greater risk of mortality); Polypharmacy = use of 5 different medications or more; GDS-15 = Geriatric Depression Scale 15 (depression with score ≥6); MMSE = Mini Mental State Examination (cognitive impairment with score ≤23); ADL-KATZ6 = Activities of Daily Living-Modified Katz Index Scale-6 (lower scores means less independency); Fatigue = NRS Fatigue, range 0–10 (higher score means worst possible fatigue); Malnourished = Short Nutritional Assessment Questionnaire (SNAQ, score ≥3); DEMMI = de Morton Mobility Index (range 0–100, higher score means better mobility skills); SPPB = Short Physical Performance Battery (range 0–12). * p-value < 0.05; Mann-Whitney U test was used for continuous variables; χ2 or Fisher’s exact test were used for categorical variables; Median Test was used Length of Stay and MSSE variable.

**Table 2:** Prevalence of decreased appetite at admission, discharge, and one and three months post-discharge.

Time Point	Prevalence Rates, % (*n*)	OR (95% CI)	*p*-Value *	Complete CasesPrevalence Rates, % (*n*)	OR (95% CI)	*p*-Value *
Admission	50.5 (202)	-	-	50.0 (99)	-	-
At discharge	34.0 (136)	0.757 (0.600–0.956)	0.019	43.4 (56)	0.768 (0.569–1.036)	0.084
One month post-discharge	27.8 (111)	0.579 (0.454–0.739)	<0.001	37.4 (74)	0.597 (0.435–0.819)	0.001
Three months post-discharge	17.0 (68)	0.359 (0.267–0.482)	<0.001	26.3 (52)	0.356 (0.245–0.519)	<0.001

* *p*-value for finding similar prevalence rates as compared to the prevalence rate at admission.

**Table 3:** Longitudinal associations of decreased appetite and muscle strength, skeletal muscle mass, mobility, and physical performance, crude and adjusted for confounders.

	Crude Model	Adjusted Model
	β (95% CI) *p*-Value	β (95% CI) *p*-Value
**Muscle Strength ** ^**1**^		
*Overall analysis*	−0.732 (−1.240; −0.224) **0.005**	−1.089 (−1.715; −0.463) **0.001**
*Hospitalzation*	−0.367 (−0.994; 0.259) 0.250	−0.840 (−1.599; −0.081) **0.030**
*Post-discharge*	−1.692 (−2.375; −1.009) **<0.001**	−1.707 (−2.559; −0.854) **<0.001**
*Admission*	−0.497 (−1.275; 0.280) 0.210	−0.934 (−1.861; −0.006) **0.049**
*At discharge*	−0.025 (−0.876; 0.827) 0.955	−0.623 (−1.675; 0.430) 0.246
*One month post-discharge*	−0.899 (−1.754; −0.045) **0.039**	−0.976 (−2.010; 0.059) 0.064
*Three months post-discharge*	−2.712 (−3.746; −1.677) **<0.001**	−2.968 (−4.351; −1.584) **<0.001**
**Skeletal Muscle Mass** ^2^		
*Overall analysis*	−0.086 (−0.545; 0.372) 0.712	−0.023 (−0.463; 0.417) 0.920
*Hospitalization*	−0.430 (−0.989; 0.129) 0.132	−0.191 (−0.724; 0.343) 0.483
*Post-discharge*	0.009 (−0.618; 0.636) 0.977	−0.006 (−0.615; 0.603) 0.984
*Admission*	−0.633 (−1.322; 0.055) 0.071	−0.382 (−1.034; 0.269) 0.250
*At discharge*	−0.209 (−0.983; 0.566) 0.597	0.056 (−0.699; 0.811) 0.884
*One month post-discharge*	0.042 (−0.726; 0.811) 0.914	0.022 (−0.722; 0.765) 0.954
*Three months post-discharge*	0.216 (−0.808; 1.239) 0.679	0.186 (−0.806; 1.177) 0.713
**Mobility** ^3^		
*Overall analysis*	−6.452 (−8.568; −4.336) **<0.001**	−3.893 (−6.061; −1.725) **<0.001**
*Hospitalization*	−5.294 (−7.847; −2.742) **<0.001**	−3.983 (-6.602; −1.364) **0.003**
*Post-discharge*	−3.061 (−5.906; −0.215) **0.035**	−1.252 (-4.262; 1.757) 0.414
*Admission*	−5.260 (−8.392; −2.129) **0.001**	−4.148 (−7.367; −0.929) **0.012**
*At discharge*	−3.787 (−7.301; −0.272) **0.035**	−3.024 (−6.678; 0.629) 0.105
*One month post-discharge*	-3.299 (-6.814; 0.215) 0.066	−2.340 (−6.060; 1.379) 0.217
*Three months post-discharge*	−1.406 (−5.740; 2.928) 0.524	1.176 (−3.635; 5.986) 0.632
**Physical performance** ^3^		
*Overall analysis*	−1.080 (−1.469; −0.692) **<0.001**	−0.706 (−1.083; −0.329) **<0.001**
*Hospitalization*	−0.770 (−1.259; −0.281) **0.002**	−0.561 (−1.021; −0.101) **0.017**
*Post-discharge*	−0.942 (−1.485; −0.399) **0.001**	−0.530 (−1.063; 0.004) 0.052
*Admission*	−0.595 (−1.199; 0.008) 0.053	−0.456 (−1.022; 0.111) 0.115
*At discharge*	−0.741 (−1.424; −0.058) **0.033**	−0.578 (−1.226; 0.071) 0.081
*One month post-discharge*	−0.873 (−1.560; −0.186) **0.013**	−0.794 (−1.459; −0.130) **0.019**
*Three months post-discharge*	−1.160 (−1.938; −0.382) **0.004**	−0.099 (−0.932; 0.734) 0.815

^1^ confounders for adjusted model: Age, gender, cognitive impairment, fatigue, depression, comorbidity and skeletal muscle mass, ^2^ confounders for adjusted model: Age, gender, cognitive impairment, fatigue, depression, comorbidity, ^3^ confounders for adjusted model: Age, gender, cognitive impairment, fatigue, depression, comorbidity and fear of falling. Bold *p*-values indicate a statistically significant difference.
